# THE INFLUENCE OF MARRIAGE AND COHABITATION ON PHYSICAL ACTIVITY AMONG MIDDLE-AGED AND OLDER PEOPLE

**DOI:** 10.1093/geroni/igac059.3004

**Published:** 2022-12-20

**Authors:** Shuhan Yuan, Kit Elam, Jeanne Johnston, Angela Chow

**Affiliations:** Indiana University, Bloomington, Bloomington, Indiana, United States; Indiana University, Bloomington, Bloomington, Indiana, United States; Indiana University Bloomington, Bloomington, Indiana, United States; Indiana University Bloomington, Bloomington, Indiana, United States

## Abstract

The Behavioral Risk Factor Surveillance System (2020) indicates that 30.6% of middle-aged and older adults were physically inactive. Whereas marital status is linked to physical activity (PA), it is increasingly common for couples to cohabitate, making it important to capture these relationships. The stress/social support theory(Burman & Margolin, 1992) highlights the importance of relationship quality in long-term relationships, but this hasn’t been cohesively examined relative to PA. This study investigated whether partnered living status (married/cohabitating) and partnered living quality (support/strain from partner, partner disagreements) were associated with PA in middle-aged/older adults. Data were from a nationally representative longitudinal study, Midlife in the United States(waves 1–3; Nf1113; aged 49–93). Subjects were categorized into four groups based on partnered living status over the three waves: partnered living at all waves (57.9%), non-partnered living (separated, divorced, widowed, or never married) at all waves (17.6%), change from partnered living to non-partnered living (20.8%), and change from non-partnered living to partnered living (3.8%). Regressions were conducted to test the effect of partnered living status and relationship quality on the frequency of moderate and vigorous PA at wave 3. Subjects who changed from non-partnered to partnered living had the highest moderate and vigorous PA levels. Partner support was positively associated with moderate PA (β=0.50, p < 0.01), and partner disagreements was negatively associated with vigorous PA (β=-0.27, p < 0.01). Results suggest that relationship status and quality can influence PA among the aging population. Public health educators should provide additional social support to older adults to promote PA.

